# Supplementary material for: The RNA-binding protein HuR is required for maintenance of the germinal centre response
Source: Nat Commun. 2021 Nov 12;12:6556. doi: 10.1038/s41467-021-26908-2 (PMC8590059; doi:10.1038/s41467-021-26908-2)
Supplement: Supplementary file 1 — Supplementary Information [file 41467_2021_26908_MOESM1_ESM.pdf]

SUPPLEMENTARY MATERIAL

The RNA-binding protein HuR is required for maintenance of the germinal centre response.

Ines C. Osma-Garcia[1], Dunja Capitan-Sobrino[1], Mailys Mouysset[1], Sarah E. Bell[2], Manuel Lebeurrier[1], Martin Turner[2] *, Manuel D. Diaz-Muñoz[1, 2] *

[1] Toulouse Institute for Infectious and Inflammatory Diseases (Infinity), Inserm UMR1291, CNRS UMR5051, University Paul Sabatier, CHU Purpan, Toulouse, France

[2] Laboratory of Lymphocyte Signalling and Development, The Babraham Institute, Cambridge, UK.

(email: martin.turner@babraham.ac.uk) (email: manuel.diaz-munoz@inserm.fr)

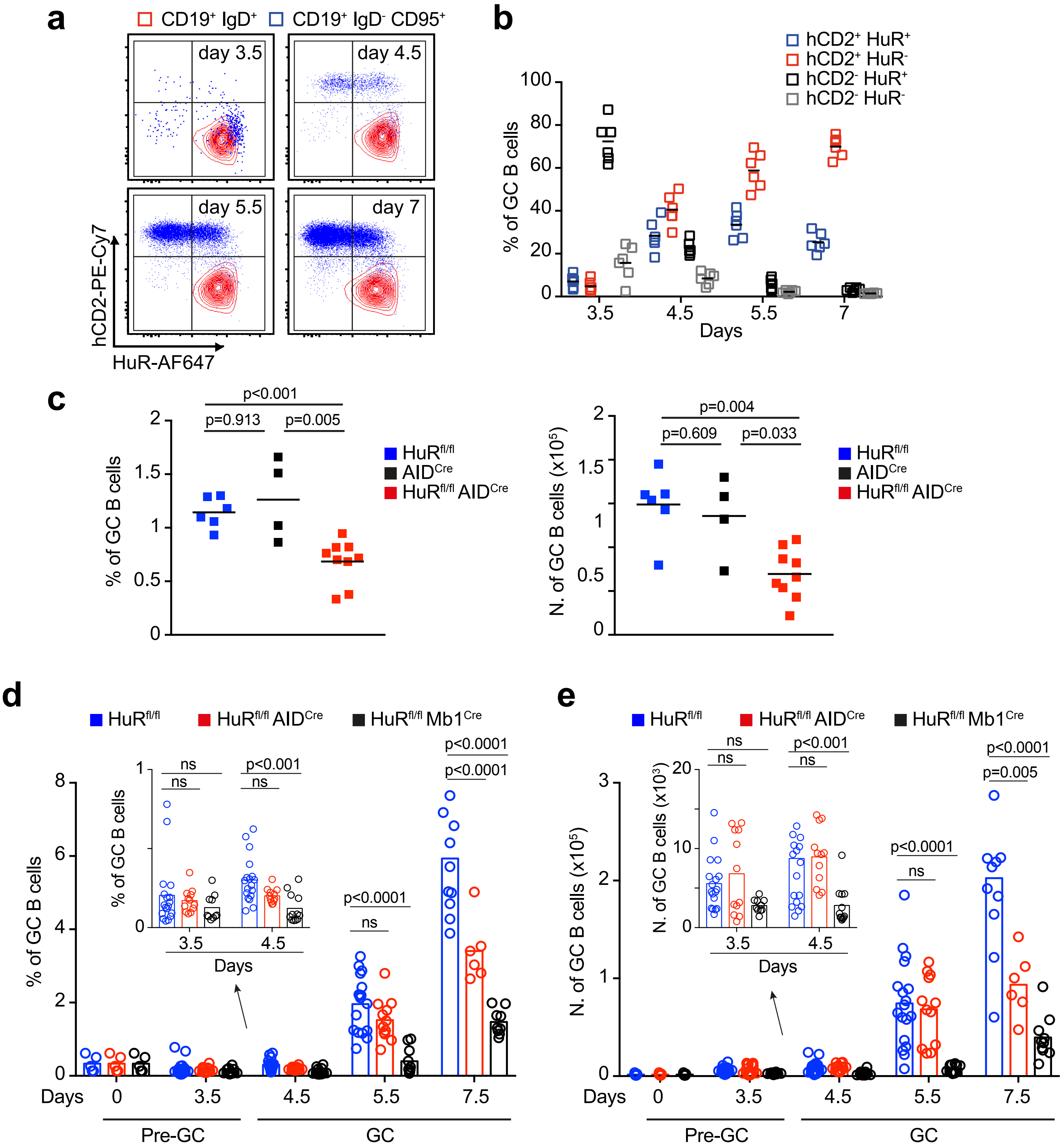
a
CD19+ IgD+
CD19+ IgD- CD95+
day 3.5
day 4.5
day 5.5
day 7
hCD2-PE-Cy7
HuR-AF647
b
hCD2+ HuR+
hCD2+ HuR-
hCD2- HuR+
hCD2- HuR-
% of GC B cells
Days
c
% of GC B cells
p<0.001
p=0.913
p=0.005
HuRfl/fl
AIDCre
HuRfl/fl AIDCre
N. of GC B cells (x10^5)
p=0.004
p=0.609
p=0.033
d
HuRfl/fl
HuRfl/fl AIDCre
HuRfl/fl Mb1Cre
% of GC B cells
Days
Pre-GC
GC
ns
p<0.001
p<0.0001
e
N. of GC B cells (x10^5)
N. of GC B cells (x10^3)
p=0.005
p<0.0001
ns
p<0.001

Supplementary Figure 1. Establishment of the GC reaction is unaffected in HuR$^{fl/fl}$ AID^{Cre} mice.

a, Representative FACs plots showing hCD2 reporter and HuR expression in CD19^{+} IgD^{+} B cells and CD19^{+} IgD^{-} CD95^{+} GC B cells in draining LNs from HuR$^{fl/fl}$ AID^{Cre} mice after s.c. hock immunisation with NP-KLH alum. **b,** Quantitation of the percentage of GC B cells expressing hCD2 and/or HuR in HuR$^{fl/fl}$ AID^{Cre} mice. Data from 1 of the 2 experiments performed, n=6 mice. **c,** Comparison of GC responses in HuR$^{fl/fl}$, AID^{Cre}, HuR$^{fl/fl}$ AID^{Cre} mice. Quantitation of the percentage (left panel) and the number (right panel) of CD19^{+} CD95^{+} GL7^{+} GC B cells in the spleen of mice at day 7 after immunisation with NP-KLH in alum. n=6 HuR$^{fl/fl}$ mice, n=4 AID^{Cre} mice and n=9 HuR$^{fl/fl}$ AID^{Cre} mice. **d,** Analysis of the percentage of GC B cells in LNs from HuR$^{fl/fl}$, HuR$^{fl/fl}$ AID^{Cre} and HuR$^{fl/fl}$ Mb1^{Cre} mice at different days after immunisation (s.c.) with NP-KLH alum. **e,** Quantitation of the number of GC B cells in mice shown in d. Data in d-e is from 3 independent experiments. n=7-18 mice per group depending on the genotype and the day of analysis, Mann-Whitney test was performed for statistical analysis in c and 2-way Anova and multiple comparison corrected using Dunnet test was performed in d and e. Source data are provided as a Source Data file.

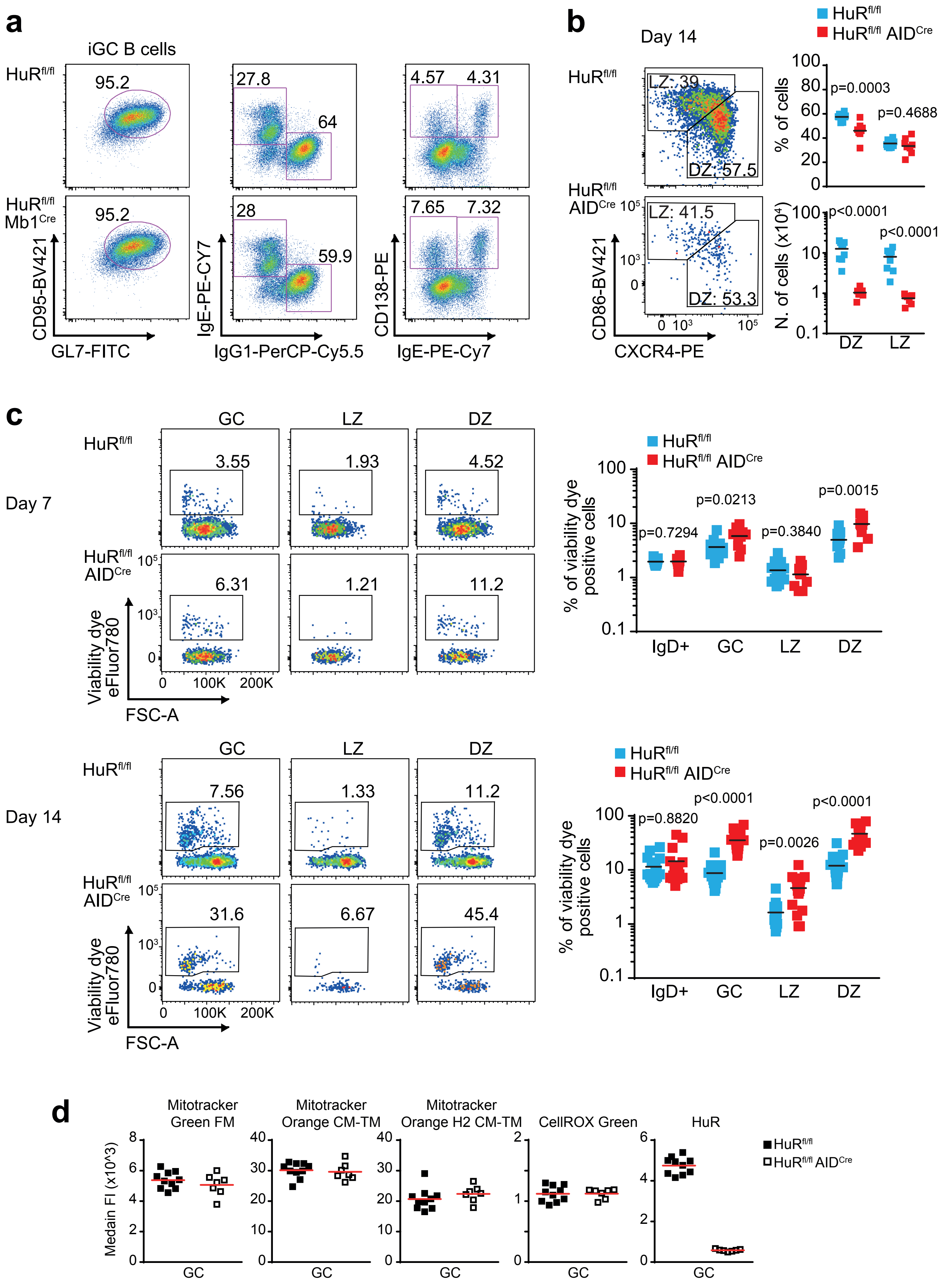
a
iGC B cells
HuRfl/fl
HuRfl/fl Mb1Cre
95.2
95.2
27.8
64
28
59.9
4.57
4.31
7.65
7.32
CD95-BV421
GL7-FITC
IgE-PE-CY7
IgG1-PerCP-Cy5.5
CD138-PE
IgE-PE-Cy7
b
Day 14
HuRfl/fl
HuRfl/fl AIDCre
LZ: 39
DZ: 57.5
LZ: 41.5
DZ: 53.3
CD86-BV421
CXCR4-PE
% of cells
p=0.0003
p=0.4688
N. of cells (x10^4)
p<0.0001
p<0.0001
DZ
LZ
c
GC
LZ
DZ
Day 7
3.55
1.93
4.52
6.31
1.21
11.2
Viability dye eFluor780
FSC-A
% of viability dye positive cells
p=0.7294
p=0.0213
p=0.3840
p=0.0015
IgD+
GC
LZ
DZ
Day 14
7.56
1.33
11.2
31.6
6.67
45.4
p=0.8820
p<0.0001
p=0.0026
p<0.0001
d
Mitotracker Green FM
Mitotracker Orange CM-TM
Mitotracker Orange H2 CM-TM
CellROX Green
HuR
Medain FI (x10^3)
GC

Supplementary Figure 2. HuR is required for survival and DZ/LZ distribution of GC B cells.

a, Phenotypic characterization of B cells co-cultured with 40LB stroma cells for 6 days in the presence of IL4 (from day 0 to day 4) and IL21 (from day 4 to day 6). Representative pseudo colour plots from 1 of the 2 independent experiments performed. FO B cells from 3 HuR$^{fl/fl}$ and 3 HuR$^{fl/fl}$ Mb1^{Cre} mice were isolated, co-cultured and analysed independently in each experiment. **b,** Analysis of DZ and LZ GC B cells in the spleens of HuR$^{fl/fl}$ and HuR$^{fl/fl}$ AID^{Cre} mice at day 14 after immunisation with NP-KLH alum. Left panels, representative contour plots of CXCR4^{+} CD86^{-} DZ B cells and CXCR4^{-} CD86^{+} LZ GC B cells (previously gated as CD19^{+} IgD^{-} CD38^{-} CD95^{+}). Right panels, percentage and number of DZ and LZ GC B cells **(**n=9 (HuR$^{fl/fl}$) and n=8 (HuR$^{fl/fl}$ AID^{Cre}) mice over 2 independent experiments, Mann-Whitney test). **c,** Representative pseudo colour plots showing staining with viability dye eFluor780 of different GC B cell populations in the spleen of HuR$^{fl/fl}$ and HuR$^{fl/fl}$ AID^{Cre} mice at day 7 and 14 after immunisation with NP-KLH alum. Right panel, quantitation of the percentage of dead B cells in control and HuR$^{fl/fl}$ AID^{Cre} mice. Data from 3 experiments performed with at least n=4 mice per group in each experiment, 2-way ANOVA and Bonferroni's post-test. **d,** Quantitation of mitochondrial mass (MitoTracker Green FM), mitochondrial membrane potential (MitoTracker Orange CM-TM), mitochondrial respiratory capacity (MitoTracker Orange H2-CM-TM) and ROS production (CellROX Green) by flow cytometry. Source data are provided as a Source Data file.

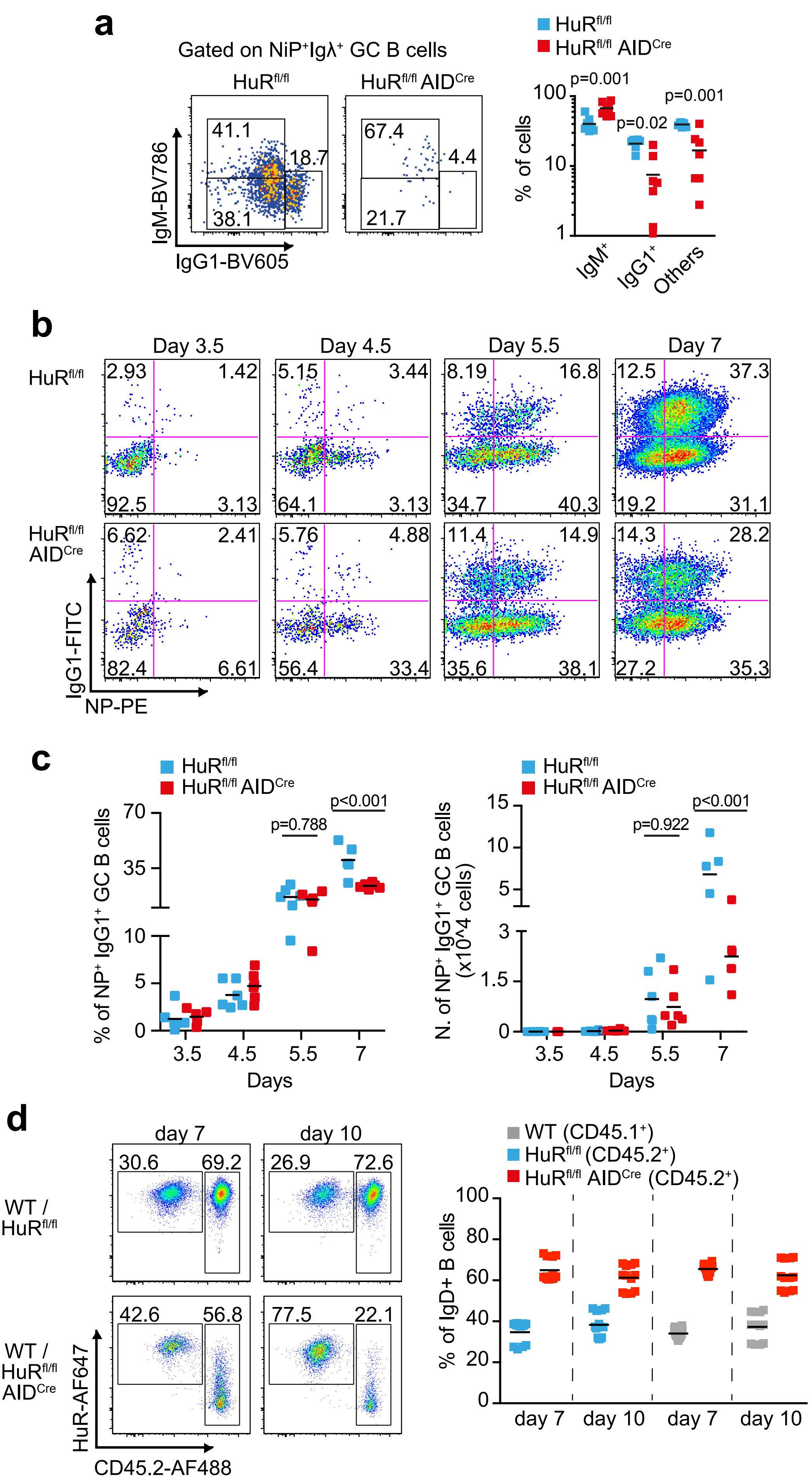
a
Gated on NiP+Igλ+ GC B cells
HuRfl/fl
HuRfl/fl AIDCre
41.1
18.7
38.1
67.4
4.4
21.7
IgM-BV786
IgG1-BV605
HuRfl/fl
HuRfl/fl AIDCre
p=0.001
p=0.02
p=0.001
% of cells
IgM+
IgG1+
Others
b
Day 3.5
Day 4.5
Day 5.5
Day 7
HuRfl/fl
HuRfl/fl AIDCre
IgG1-FITC
NP-PE
c
HuRfl/fl
HuRfl/fl AIDCre
p=0.788
p<0.001
% of NP+ IgG1+ GC B cells
Days
p=0.922
p<0.001
N. of NP+ IgG1+ GC B cells (x10^4 cells)
Days
d
day 7
day 10
WT / HuRfl/fl
WT / HuRfl/fl AIDCre
HuR-AF647
CD45.2-AF488
WT (CD45.1+)
HuRfl/fl (CD45.2+)
HuRfl/fl AIDCre (CD45.2+)
% of IgD+ B cells
day 7
day 10
day 7
day 10

Supplementary Figure 3. HuR is dispensable for the appearance of antigen-specific GC B cells.

a, Analysis of the Ig isotype of Igλ^+ NiP$^+$ GC B cells found in the spleen of HuR$^{fl/fl}$ and HuR$^{fl/fl}$ AID^{Cre} mice at day 14 after immunisation with NP-KLH in alum. Left panels, representative pseudo colour plots of IgM and IgG1 expression by Igλ^+ NiP$^+$ GC B cells (previously gated on CD19$^+$ IgD$^-$ CD38$^-$ CD95$^+$). Right panels, percentage of IgM$^+$, IgG1$^+$ and IgM$^-$ IgG1$^-$ (others) GC B cells (data representative from 1 of the 2 independent experiments performed, n=7 mice per group, Mann-Whitney test). **b,** Analysis of NP- binding GC B cells (gated as CD19$^+$ IgD$^-$ CD95$^+$ cells) in draining LNs of of HuR$^{fl/fl}$ and HuR$^{fl/fl}$ AID^{Cre} mice after immunisation with NP-KLH in alum. Representative dot plots from 1 of the 2 independent experiment performed are shown. **c,** Quantitation of the percentage (left panel) and the number (right panel) of NP+ IgG1+ GC B cells shown in b. Data are representative from 1 of the 2 experiments performed, n=5 or 6 mice per group depending on the genotype and the day of analysis (2-way ANOVA and Bonferroni's post-test). **d,** Analysis of the proportion of CD45.2- and CD45.2+ B cells (gated as CD19+ IgD-) in draining LNs of BM chimera mice (WT CD45.1+ / HuR$^{fl/fl}$ CD45.2+ or CD45.1+ / HuR$^{fl/fl}$ AID^{Cre} CD45.2+, ratio 1:3) shown in Figure 3c. Data are from n=12 or 16 mice per group depending on the genotype and the day of analysis. Source data are provided as a Source Data file.

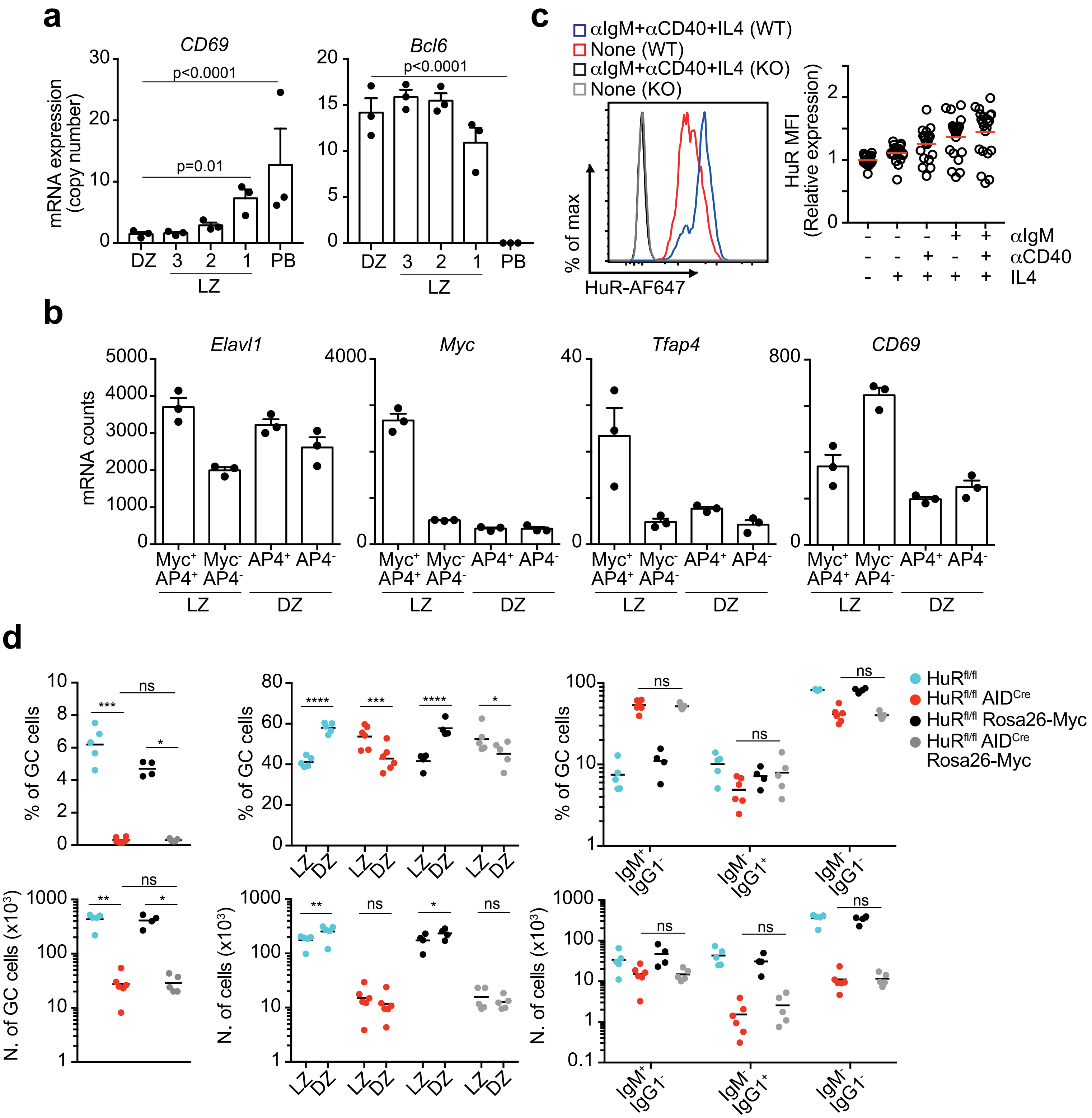

a
CD69
mRNA expression (copy number)
p<0.0001
p=0.01
DZ 3 2 1 PB
LZ
Bcl6
p<0.0001
b
Elavl1
Myc
Tfap4
CD69
mRNA counts
Myc+ AP4+
Myc- AP4-
AP4+
AP4-
LZ
DZ
c
αIgM+αCD40+IL4 (WT)
None (WT)
αIgM+αCD40+IL4 (KO)
None (KO)
% of max
HuR-AF647
HuR MFI (Relative expression)
αIgM
αCD40
IL4
d
% of GC cells
N. of GC cells (x10³)
N. of cells (x10³)
LZ DZ
IgM+ IgG1-
IgM- IgG1+
IgM- IgG1-
ns
HuRfl/fl
HuRfl/fl AIDCre
HuRfl/fl Rosa26-Myc
HuRfl/fl AIDCre Rosa26-Myc

Supplementary Figure 4. Myc over-expression does not rescue GCs in HuR$^{fl/fl}$ AID^{Cre} mice.

a, *CD69* and *Bcl6* mRNA expression in the cell populations described in figure 4a (n=3 samples per condition, DESeq2 analysis, BH correction of p-values). **b,** *Elavl1*, *Myc*, *Tfap4* and *CD69* mRNA levels in selected (Myc^{+} AP4^{+}) and non-selected (Myc^{-} AP4^{-}) LZ GC B cells by Tfh cells; and in AP4^{+}and AP4^{-} DZ B cells (GSE80669 (Chou et al., 2016), n=3 samples per group, mean±SD, DESeq2 analysis). Data in a-b are presented as mean values +/- SEM. **c,** Expression of HuR in splenic WT FO B cells stimulated or not with IL-4, αCD40+IL4, αIgM+IL4 or αCD40+αIgM+IL4 with the mitogens and cytokines indicated (n=22 mice from 5 independent experiments performed each with at least n=3 biological replicates per group). **d,** Phenotypic characterization of GC responses in B6.SJL mice reconstituted with HuR$^{fl/fl}$, HuR$^{fl/fl}$ AID^{Cre} , HuR$^{fl/fl}$ Rosa26-Myc and HuR$^{fl/fl}$ Rosa26-Myc AID^{Cre} bone marrow at day 14 after immunisation with NP-KLH (n=6 for mouse groups HuR$^{fl/fl}$, HuR$^{fl/fl}$ AID^{Cre} and HuR$^{fl/fl}$ Rosa26-Myc, and n=4 for HuR$^{fl/fl}$ Rosa26-Myc AID^{Cre} mouse group, Mann-Whitney test, *p<0.05, **p<0.01, ***p<0.001, ****p<0.0001). Source data are provided as a Source Data file.

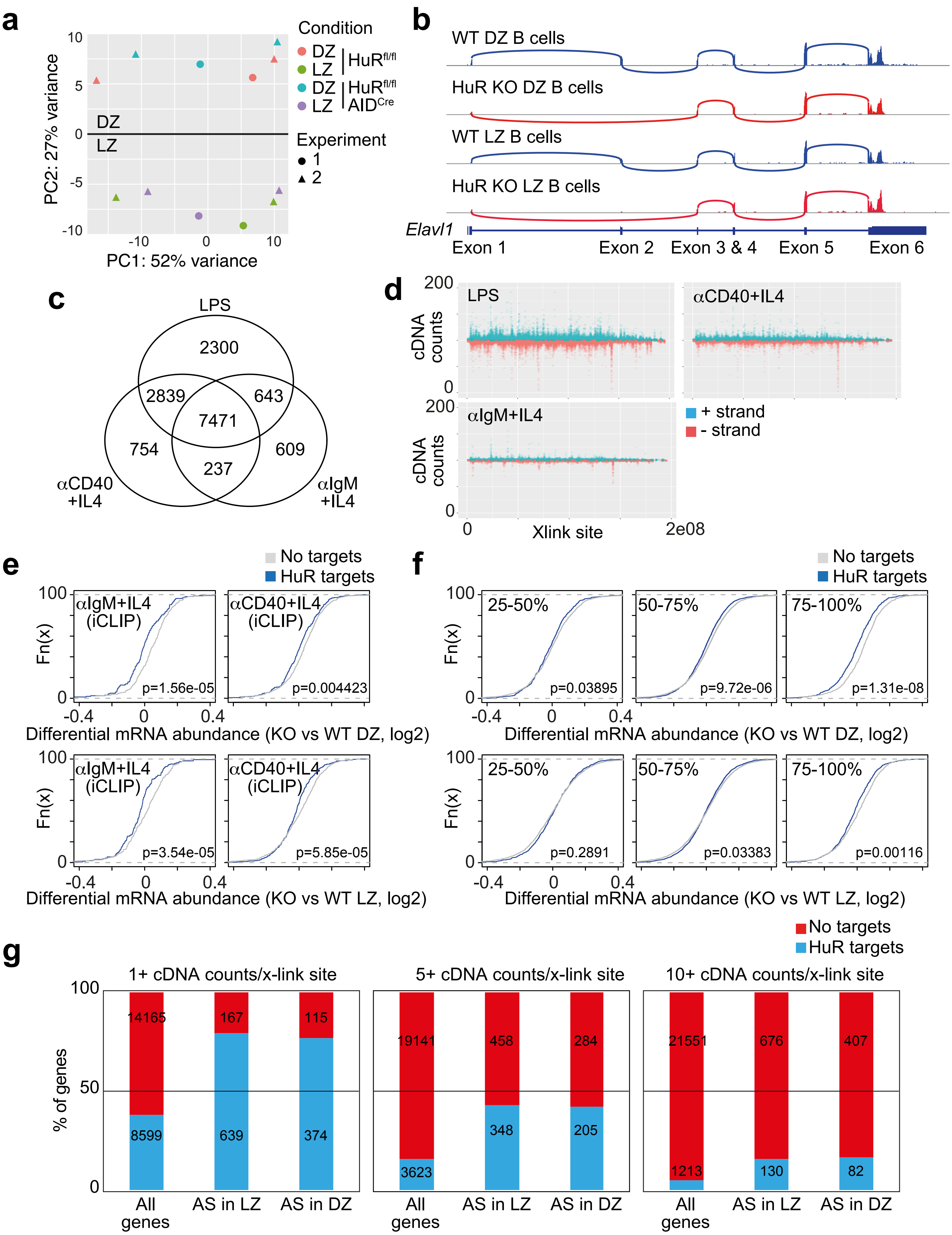

a
Condition
DZ
LZ
HuR fl/fl
DZ
LZ
HuR fl/fl AID Cre
Experiment
1
2
DZ
LZ
PC2: 27% variance
PC1: 52% variance
b
WT DZ B cells
HuR KO DZ B cells
WT LZ B cells
HuR KO LZ B cells
Elavl1
Exon 1
Exon 2
Exon 3 & 4
Exon 5
Exon 6
c
LPS
2300
2839
643
7471
754
609
237
αCD40 +IL4
αIgM +IL4
d
LPS
αCD40+IL4
αIgM+IL4
cDNA counts
+ strand
- strand
Xlink site
2e08
e
No targets
HuR targets
αIgM+IL4 (iCLIP)
αCD40+IL4 (iCLIP)
p=1.56e-05
p=0.004423
Fn(x)
Differential mRNA abundance (KO vs WT DZ, log2)
p=3.54e-05
p=5.85e-05
Differential mRNA abundance (KO vs WT LZ, log2)
f
No targets
HuR targets
25-50%
50-75%
75-100%
p=0.03895
p=9.72e-06
p=1.31e-08
Differential mRNA abundance (KO vs WT DZ, log2)
p=0.2891
p=0.03383
p=0.00116
Differential mRNA abundance (KO vs WT LZ, log2)
g
No targets
HuR targets
1+ cDNA counts/x-link site
5+ cDNA counts/x-link site
10+ cDNA counts/x-link site
% of genes
14165
8599
167
639
115
374
19141
3623
458
348
284
205
21551
1213
676
130
407
82
All genes
AS in LZ
AS in DZ

Supplementary Figure 5. Transcriptomics analysis of DZ and LZ GC HuR KO B cells.

a, Principal component analysis of mRNAseq data from DZ and LZ GC B cells sorted from $HuR^{fl/fl}$ (WT) and $HuR^{fl/fl}$ AID^{Cre} (HuR KO) mice at day 7 after immunisation with NP-KLH in alum (DESeq2). **b,** Visualization as Sashimi plot of exon-exon spanning reads mapped to the *Elavl1* (HuR) gene. **c,** Venn diagram showing the overlap of genes which mRNAs were identified as targets of HuR in iCLIP experiments performed upon B cell stimulation for 48 h. with LPS, αCD40+IL4 or αIgM+IL4. **d,** iCLIP data depth represented as the number of unique cDNA counts annotated to each of the HuR crosslink sites identified in the iCLIP assays shown in c. **e,** Cumulative distribution of differential mRNA abundance in DZ KO and LZ KO GC B cells compared to WT cells of HuR mRNA targets and mRNAs not bound with HuR. All mRNA transcripts were merged by gene. Data is divided based on HuR mRNA target detection in the transcript 3'UTR (at least 5- unique cDNA counts per x-link site) in HuR iCLIP assays from B cells stimulated with αCD40+IL4 or αIgM+IL4. Data from the top 25% of most expressed genes is shown as in figure 5d (Kolmogorov–Smirnov test). **f,** Cumulative distribution of differential mRNA abundance upon gene clustering based on the expression of HuR mRNA targets to account for iCLIP experimental bias (iCLIP preferential detection of highly expressed genes). Changes in mRNA abundance were subdivided and plotted in equal windows having each a 25% of genes. Data is shown from the less abundant (25-50% window) to the most abundant (75-100% window) (HuR binding was annotated if at least 5- unique cDNA counts per x-link site were detected) (Kolmogorov–Smirnov test). **g,** Percentage and number of genes annotated as alternatively spliced in HuR KO GC B cells that are also targets of HuR. Data were classified based on the number of cDNA counts annotated in each crosslink site.

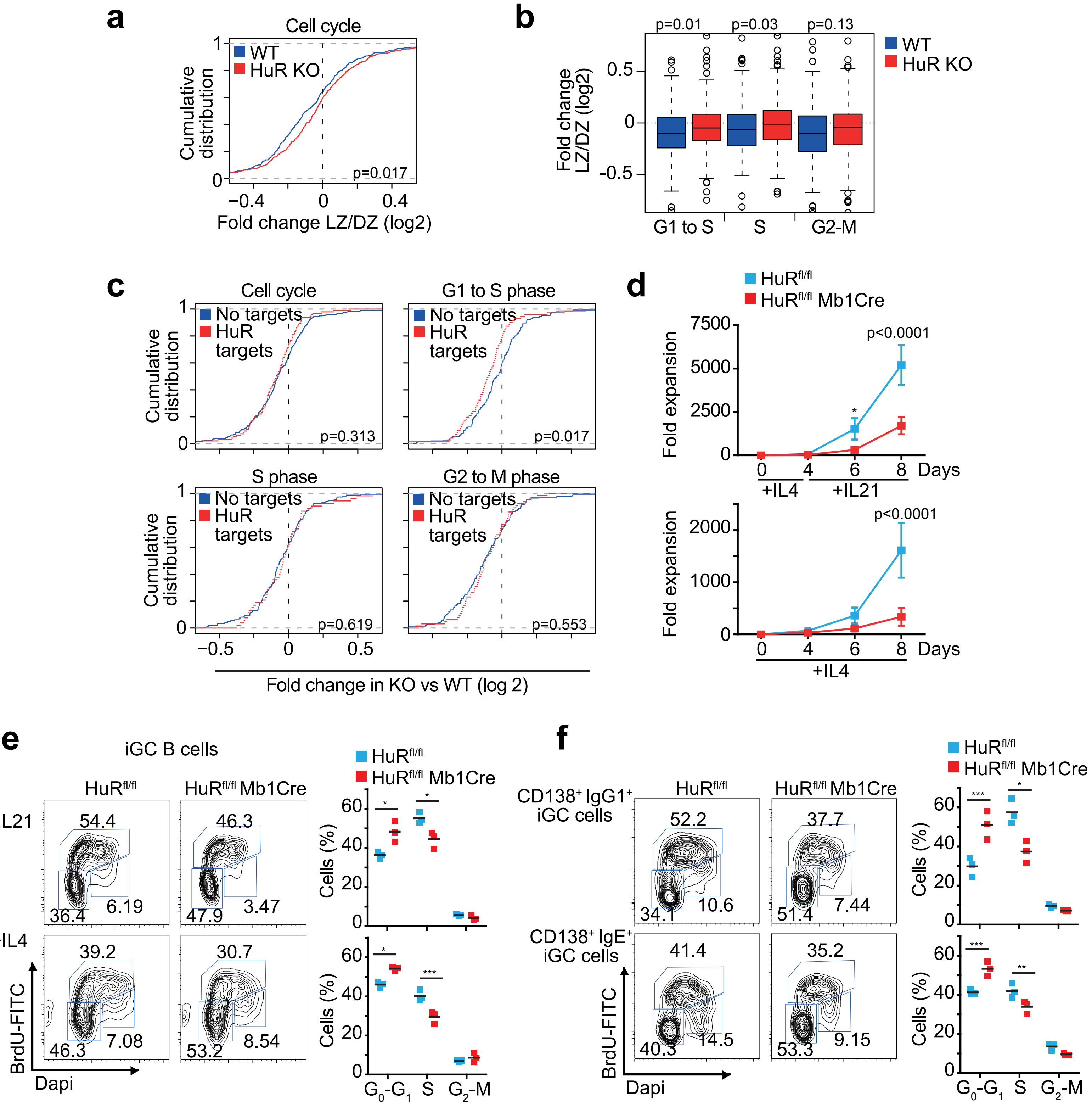
a
Cell cycle
WT
HuR KO
Cumulative distribution
p=0.017
Fold change LZ/DZ (log2)
b
p=0.01
p=0.03
p=0.13
WT
HuR KO
Fold change LZ/DZ (log2)
G1 to S
S
G2-M
c
Cell cycle
G1 to S phase
S phase
G2 to M phase
No targets
HuR targets
Cumulative distribution
p=0.313
p=0.017
p=0.619
p=0.553
Fold change in KO vs WT (log 2)
d
HuRfl/fl
HuRfl/fl Mb1Cre
Fold expansion
p<0.0001
Days
+IL4
+IL21
e
iGC B cells
HuRfl/fl
HuRfl/fl Mb1Cre
+IL21
+IL4
BrdU-FITC
Dapi
Cells (%)
G0-G1
S
G2-M
f
CD138+ IgG1+ iGC cells
CD138+ IgE+ iGC cells
HuRfl/fl
HuRfl/fl Mb1Cre
BrdU-FITC
Dapi
Cells (%)

Supplementary Figure 6. HuR controls cell cycle.

a, Cumulative distribution of change in expression of genes associated to the cell cycle in LZ and DZ GC B cells (Kolmogorov–Smirnov test). **b**, Boxplots showing changes in the expression of cell cycle genes classified into G1-early S (n=216 genes), S (n=201 genes) and G2-M (n=207 genes) phases of the cell cycle (n=436 genes, data show the mean, 25-75% range and 5-95% whiskers. Wilcox test). **c,** Cumulative distribution of change in expression in DZ GC B cells of HuR-target genes or not associated to G1-S, S and G2-M phases of the cell cycle (Kolmogorov–Smirnov test). **d,** Expansion of $HuR^{fl/fl}$ and $HuR^{fl/fl}$ $Mb1^{Cre}$ iGC B cells cultured in-vitro for 8 days (representative data from 1 of 3 independent experiments, n=3 mice per group, Data presented as mean values +/- SD, 2-way ANOVA and Tukey post-test, * p<0.05, *** p<0.001). **e,** BrdU incorporation in proliferating iGC B cells at day 6 of culture. IGC B cells were supplemented with IL-4 or IL-21. Left panels, representative contour plots of BrdU and DNA content (Dapi) staining. Right panels, percentage of cells in G0-G1, S and G2-M phases of the cell cycle (representative data from 1 of the 3 independent experiments performed, n=3 mice per group, 2-way ANOVA and Tukey post- test, * p<0.05, *** p<0.001). **f,** BrdU incorporation in proliferating $CD138^{+}$ iGC B cells at day 6. Left panels, representative contour plots of BrdU and DNA content (Dapi) staining. Right panels, percentage of cells in G0-G1, S and G2-M phases (representative data from 1 of the 3 independent experiments performed, n=3 mice per group, 2-way ANOVA and Tukey post- test, * p<0.05, *** p<0.001). Source data are provided as a Source Data file.

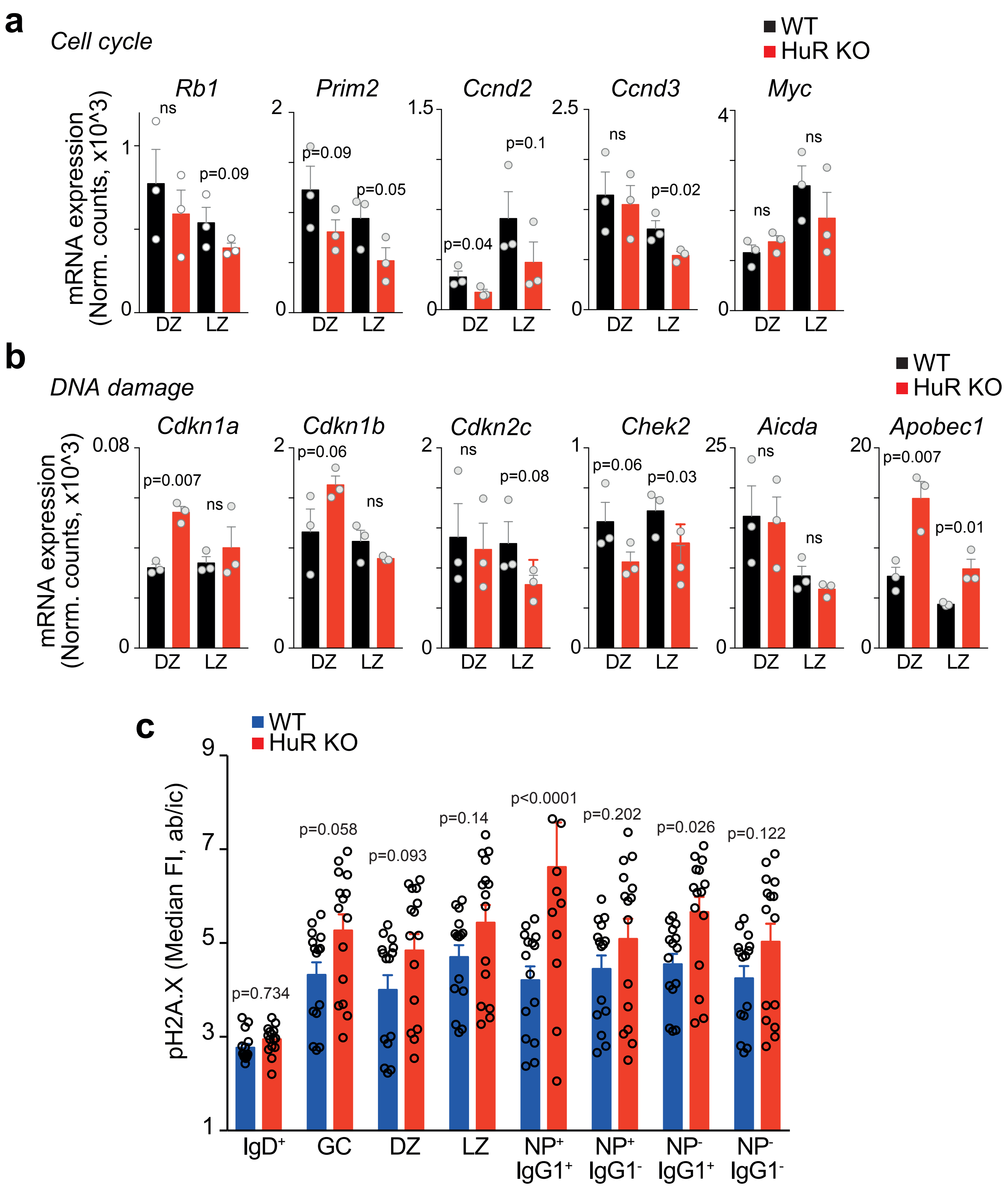

a
Cell cycle
WT
HuR KO
Rb1
Prim2
Ccnd2
Ccnd3
Myc
mRNA expression
(Norm. counts, x10^3)
ns
p=0.09
p=0.09
p=0.05
p=0.04
p=0.1
ns
p=0.02
ns
ns
DZ
LZ
b
DNA damage
WT
HuR KO
Cdkn1a
Cdkn1b
Cdkn2c
Chek2
Aicda
Apobec1
p=0.007
ns
p=0.06
ns
ns
p=0.08
p=0.06
p=0.03
ns
ns
p=0.007
p=0.01
c
WT
HuR KO
pH2A.X (Median FI, ab/ic)
p=0.734
p=0.058
p=0.093
p=0.14
p<0.0001
p=0.202
p=0.026
p=0.122
IgD+
GC
DZ
LZ
NP+ IgG1+
NP+ IgG1-
NP- IgG1+
NP- IgG1-

Supplementary Figure 7. H2A.X phosphorylation is increased in the absence of HuR.
a, Quantitation of the mRNA expression of *Rb1*, *Prim2*, *Ccnd2*, *Ccnd3* and *Myc* in DZ and LZ GC B cells. **b,** Analysis of the expression of DNA damage associated genes *Cdkn1a*, *Cdkn1b*, *Cdkn2c*, *Chk2*, *Aicda* and *Apobec1*. Data in a and b are collected from n=3 independent samples analysed by mRNAseq and DESeq2 (BH method for adjusted p-value calculation) and presented as mean values +/- SEM. **c,** Median fluorescence intensity of pH2A.X (pS139) shown in Figure 7g. Data from n=15 mice per group collected over 2 independent experiments. Mean ± SEM is shown. Mann-Whitney test.

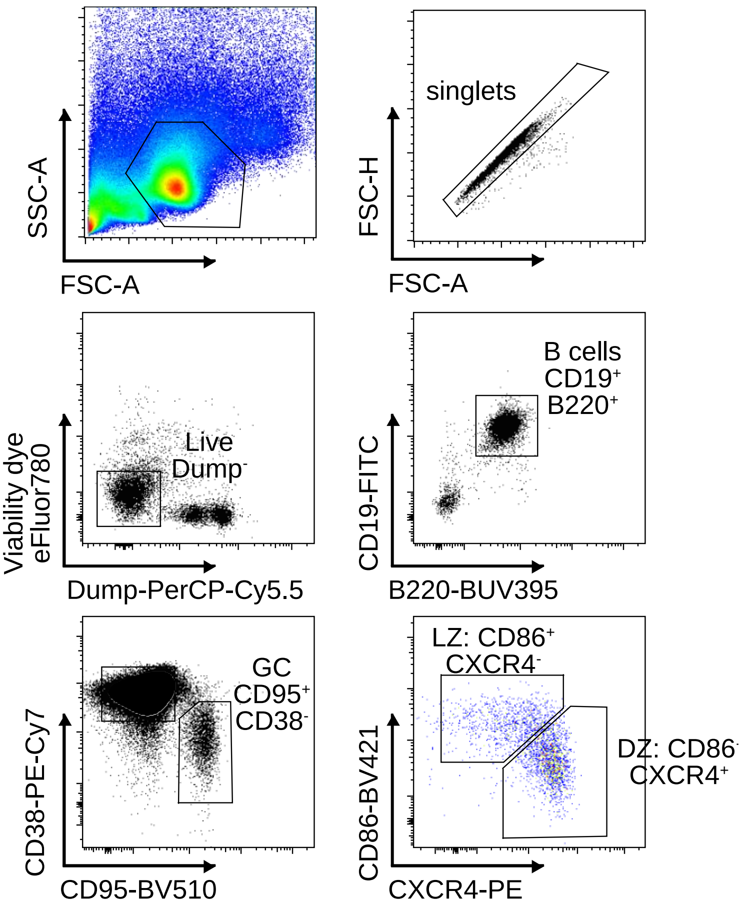

Supplementary Figure 8. Gating strategy for FACs sorting of DZ and LZ GC B cells for RNAseq.

Supplementary Table 1. List of antibodies.

Protein detected	Source	Ig type (clone)	Application	Dilution	Company	Cat. Numbers.
HuR	Mouse	IgG1 (3A2)	FC	1:1000	Santa Cruz	sc-5261
Isotype ab	Mouse	IgG1 (G3A1)	FC	1:1000	Cell Signaling Tech	5415S
B-actin	Mouse	IgG1 (AC-15)	WB	1:10000	Sigma	A1978
Myc	Rabbit	IgG (D84C12)	FC/WB	1:100	Cell Signaling Tech	5605 and 13871
Rabbit Ig	Goat	IRDye 680 conjugated	WB	1:10000	LI-COR Biosciences	P/N 925-68073
Mouse Ig	Goat	IRDye 800CW conjugated	WB	1:10000	LI-COR Biosciences	P/N 925-32212
B220	Rat	IgG2a (RA3-6B2)	FC	1:400	BD Biosciences	103206
CD19	Rat	IgG2a (6D5)	FC	1:400	BioLegend	115505
IgD	Rat	IgG2a (11-26.2a)	FC/IF	1:400	BioLegend	405718
IgM	Rat	IgG2a (R6-60.2)	FC	1:100	BD Biosciences	550881 and 564028
IgE	Rat	IgG1 (RME-1)	FC	1:400	BioLegend	406903
IgG1	Rat	IgG1 (RMG1-1)	FC	1:500	BioLegend	406615
Ig light chain λ	Rat	IgG (RML-42)	FC	1:400	BioLegend	407307
CD95	Hamster	IgG2 (Jo2)	FC	1:400	BD Biosciences	563646
GL7	Rat	IgM (GL7)	FC	1:200	BioLegend	144603
CXCR4	Rat	IgG2b (2B11)	FC	1:100	BD Biosciences	551966
CD38	Rat	IgG2a (90)	FC	1:400	BD Biosciences	562770
CD86	Rat	IgG2a (GL1)	FC	1:400	BD Biosciences	564198
CD45.2	Mouse	IgG2a (104)	FC	1:400	BioLegend	109806
CD2	Mouse	IgG1 (RPA-2.10)	FC	1:200	BioLegend	300203
CD138	Rat	IgG2a (7E9)	FC	1:800	BD Biosciences	553714
CD21/35	Rat	IgG2a (281-2)	IF	1:400	BioLegend	123405
Ki67	Rat	IgG2a (SolA15)	IF	1:400	ThermoScientific	50-5698-80
IgM	Rat	IgG2a (II/41)	Elisa	1:1000	BD Biosciences	553435
IgG1	Rat	IgG1 (A85-3)	Elisa	1:1000	BD Biosciences	553445
IgM	Rat	IgG2a (R6-60.2)	Elisa	1:1000	BD Biosciences	553405
IgG1	Rat	Rat IgG1(A85-1)	Elisa	1:1000	BD Biosciences	553440
IgM	Goat	IgG polyclonal	Elisa	1:1000	Southern Biotech	3020-08
IgG (H+L)	Goat	IgG polyclonal	Elisa	1:1000	Southern Biotech	3050-08
GFP	Rabbit	IgG polyclonal	FC	1:500	ThermoScientific	A21311
phosphoSer139-H2a.X	Mouse	IgG1(clone 2F3)	FC	1:100	BioLegend	613406
NP-PE	-	-	FC	1:1000	Biosearch Tech	N-5070-1-BS
NiP-BSA-Biotin	-	-	FC	1:1000	Biosearch Tech	N-1027-5
BrdU	Mouse	IgG1 (MoBU-1)	FC	1:200	ThermoScientific	B35133
CaspACE™ FITC-VAD-FMK In Situ Marker	-	-	FC	1 mM	Promega	G7461
CellROX™ Reagent Variety Pack	-	-	FC	1 mM	ThermoScientific	C10448
MitoTracker™ Orange CMTMRos	-	-	FC	1 mM	ThermoScientific	M7510
MitoTracker™ Orange CM-H2TMRos	-	-	FC	1 mM	ThermoScientific	M7511
Molecular Probes MitoTracker Green FM	-	-	FC	1 mM	ThermoScientific	M7514

Supplementary Table 2. List of primers.

Name	Sequence	Target gene/region	Orientation	Application
JH4 forw	CCTAGGAACCAACTTAAGAGT	Ig JH4 region	Forward	PCR
JH4 rev	TGGAGTTTTCTGAGCATTGCAG	Ig JH4 region	Reverse	PCR
