# Supplementary material for: The RNA-binding protein HuR is required for maintenance of the germinal centre response
Source: Nat Commun. 2021 Nov 12;12:6556. doi: 10.1038/s41467-021-26908-2 (PMC8590059; doi:10.1038/s41467-021-26908-2)
Supplement: Supplementary file 3 — Description of Additional Supplementary Files [file 41467_2021_26908_MOESM3_ESM.pdf]

Description of Additional Supplementary Files

File Name: Supplementary Data 1
Description: mRNAseq analysis by DESeq2.

File Name: Supplementary Data 2
Description: RNA splicing analysis with rMATS.

File Name: Supplementary Data 3
Description: Gene ontology analysis with Toppgene.

File Name: Supplementary Data 4
Description: Curated list of cell cycle genes (from Reactome and (Macosko et al., 2015) classified based on their expression in G1-early S, S or G2-M phases of the cell cycle.
