# Supplementary material for: The RNA-binding protein HuR is required for maintenance of the germinal centre response
Source: Nat Commun. 2021 Nov 12;12:6556. doi: 10.1038/s41467-021-26908-2 (PMC8590059; doi:10.1038/s41467-021-26908-2)
Supplement: Supplementary file 8 — Reporting Summary [file 41467_2021_26908_MOESM8_ESM.pdf]

nature research

Corresponding author(s): Manuel D. Diaz-Munoz

Last updated by author(s): Sep 22, 2021

Reporting Summary

Nature Research wishes to improve the reproducibility of the work that we publish. This form provides structure for consistency and transparency in reporting. For further information on Nature Research policies, see our Editorial Policies and the Editorial Policy Checklist.

Statistics

For all statistical analyses, confirm that the following items are present in the figure legend, table legend, main text, or Methods section.

n/a	Confirmed	
☐	☒	The exact sample size (*n*) for each experimental group/condition, given as a discrete number and unit of measurement
☐	☒	A statement on whether measurements were taken from distinct samples or whether the same sample was measured repeatedly
☐	☒	The statistical test(s) used AND whether they are one- or two-sided<br>*Only common tests should be described solely by name; describe more complex techniques in the Methods section.*
☐	☒	A description of all covariates tested
☐	☒	A description of any assumptions or corrections, such as tests of normality and adjustment for multiple comparisons
☐	☒	A full description of the statistical parameters including central tendency (e.g. means) or other basic estimates (e.g. regression coefficient) AND variation (e.g. standard deviation) or associated estimates of uncertainty (e.g. confidence intervals)
☐	☒	For null hypothesis testing, the test statistic (e.g. *F*, *t*, *r*) with confidence intervals, effect sizes, degrees of freedom and *P* value noted<br>*Give P values as exact values whenever suitable.*
☒	☐	For Bayesian analysis, information on the choice of priors and Markov chain Monte Carlo settings
☒	☐	For hierarchical and complex designs, identification of the appropriate level for tests and full reporting of outcomes
☒	☐	Estimates of effect sizes (e.g. Cohen's *d*, Pearson's *r*), indicating how they were calculated

Our web collection on statistics for biologists contains articles on many of the points above.

Software and code

Policy information about availability of computer code

Data collection	Data was collected in a BD Fortessa using the BD FACSDiva™ Software. Sequencing data was collected using the Illumina HiSeq 2500 platform. Imaging data was collected in a Leica SP8 confocal microscope using Leica Application Suite X (LAS X) software.
Data analysis	FACs data was analysed with FlowJo vX software. Confocal images were analysed with FIJI Image J software.<br>Bioinformatics data analyses were performed with TrimGalore (v0.4.4), HiSat (v2.1.0), SamTools (v1.8), FeatureCounts (v1.6.3), DESeq2 (v1.24.0), rMATS (v4.0.2), Flexbar (v3.0), iCount pipeline (https://icount.readthedocs.io/), R (v3.4.1), bioMart (v2.40.4), GenomicRanges (v1.36.0), ToppGene, GSEA vs.3 and Mus_musculus.GRCm38.90.gtf. Data was plotted in R (v3.4.1) using ggplot2 (v3.2.1).

For manuscripts utilizing custom algorithms or software that are central to the research but not yet described in published literature, software must be made available to editors and reviewers. We strongly encourage code deposition in a community repository (e.g. GitHub). See the Nature Research guidelines for submitting code & software for further information.

Data

Policy information about availability of data

All manuscripts must include a data availability statement. This statement should provide the following information, where applicable:

- Accession codes, unique identifiers, or web links for publicly available datasets
- A list of figures that have associated raw data
- A description of any restrictions on data availability

Raw data from RNAseq datasets presented in Figures 5, 6, 7 and 8 is deposited in Gene Omnibus (GEO), accession code GSE145413. Additional data in Figure 4 is from previously published RNAseq datasets and deposited in GEO with the accession numbers GSE109732, GSE80669 and GSE82003. iCLIP data was published and deposited in GEO with the accession number GSE62148.

Field-specific reporting

Please select the one below that is the best fit for your research. If you are not sure, read the appropriate sections before making your selection.

☒ Life sciences ☐ Behavioural & social sciences ☐ Ecological, evolutionary & environmental sciences

For a reference copy of the document with all sections, see nature.com/documents/nr-reporting-summary-flat.pdf

Life sciences study design

All studies must disclose on these points even when the disclosure is negative.

Sample size	The number of animals was decided on the basis of preliminary data and statistical power calculations. Data fulfil a log distribution, the null hypothesis is that the medians of the two samples are identical and assuming a 2-fold difference between means, a standard deviation of 1/3 of the mean value, a ratio between groups of 1 and a type I error rate of 5% we need a minimum of 6-7 mice per group and experiment for an 90% statistical power.
Data exclusions	No animals were excluded due to a lack of responsiveness to immunisation.
Replication	Experiments were replicated successfully a minimum of 2-3 times. Independent data points are presented in those relevant figure.
Randomization	Randomization was set in these studies by housing control and experimental mice together from birth. Breeding strategy was set to generate control and experimental mice from the same litter in 50%-50% proportions. Allocation of mice into experimental groups from different litters only considered matching the age of mice (8-16 weeks old).
Blinding	No blinding was performed as the strong phenotype of conditional KO mice allowed easy discrimination between genotypes.

Reporting for specific materials, systems and methods

We require information from authors about some types of materials, experimental systems and methods used in many studies. Here, indicate whether each material, system or method listed is relevant to your study. If you are not sure if a list item applies to your research, read the appropriate section before selecting a response.

Materials & experimental systems

n/a	Involved in the study	
☐	☒	Antibodies
☒	☐	Eukaryotic cell lines
☒	☐	Palaeontology and archaeology
☐	☒	Animals and other organisms
☒	☐	Human research participants
☒	☐	Clinical data
☒	☐	Dual use research of concern

Methods

n/a	Involved in the study	
☒	☐	ChIP-seq
☐	☒	Flow cytometry
☒	☐	MRI-based neuroimaging

Antibodies

Antibodies used	Described in supplementary table 1.
Validation	Antibody against HuR was validated using HuR KO B cells previously (Figure 1. Diaz-Munoz et al. Nat Immunol. 2015 Apr; 16(4): 415–425; doi: 10.1038/ni.3115). Standardized antibodies routinarely validated by BD Biosciences or BioLegend were used in flow cytometry analyses.

Animals and other organisms

Policy information about studies involving animals; ARRIVE guidelines recommended for reporting animal research

Laboratory animals	Elavl1tm1dkon (HuRfl/fl), Tg(Aicda-cre)9Mbu (AIDCre), CD79atm1(cre)Reth (Mb1Cre), Gt(ROSA)26Sortm13(CAG-MYC,-CD2*)Rsky B6.Rag2tm1.1Cgn (Rag2-KO) and C57BL/6-Ly5.1 (CD45.1+) mice. All strains were kept in a C57BL/6 background, males and females (8–16 weeks old) were used in this study.
Wild animals	No wild animals were used in this study.
Field-collected samples	No field-collected samples were used in the study.
Ethics oversight	All procedures with mice were approved by the Babraham Institute (UK) and the Toulouse Institute for Infectious and Inflammatory

Ethics oversight	Diseases and CREFRE local ethical committees as well as by the UK Home Office and the French Ministry of Education, Research and Innovation.

Note that full information on the approval of the study protocol must also be provided in the manuscript.

Flow Cytometry

Plots

Confirm that:

☒ The axis labels state the marker and fluorochrome used (e.g. CD4-FITC).

☒ The axis scales are clearly visible. Include numbers along axes only for bottom left plot of group (a 'group' is an analysis of identical markers).

☒ All plots are contour plots with outliers or pseudocolor plots.

☒ A numerical value for number of cells or percentage (with statistics) is provided.

Methodology

Sample preparation	Single cells suspensions were generated from spleen or peripheral lymph nodes (axilar, brachial and inguinal) by smashing tissues using cell strainers. Cells were resuspended in PBS containing 2% fetal calf serum for cell staining with the selected antibodies.
Instrument	FACs data was collected in a BD Fortessa. Sequencing data was collected using the Illumina HiSeq 2500 platform. Imaging data was collected in a Leica SP8 confocal microscope.
Software	FACs data was analysed with FlowJo vX software. Confocal images were analysed with FIJI Image J software.
Cell population abundance	The percentage of Germinal centre B cells (60% dark zone GC B cells and 40% LZ GC B cells) varies between 1-3% of total B cells in spleen at day 7 post-immunisation. DZ and LZ B cells were sorted with over a 90% purity
Gating strategy	Normal gating strategy was as follow: 1 - Lymphocytes based on FSC-A/SSC-A, 2 - singlets based on FSC-A/FSC-H, 2 - singlets based on FSC-A/FSC-W, 4 - activated B cells based on CD19+ IgD+, 5 - germinal centre B cells based on CD95+ CD38-, 6 - dark zone/light zone based on CXCR4 and CD86.<br>As summary: GC B cells - CD19+ IgD- CD38- CD95+, DZ GC B cells - CD19+ IgD- CD38- CD95+ CXCR4+ CD86-. LZ GC B cells - CD19+ IgD- CD38- CD95+ CXCR4- CD86+.

☒ Tick this box to confirm that a figure exemplifying the gating strategy is provided in the Supplementary Information.
